# Advances in m6A modification in pregnancy-related disorders and endocrine diseases of the female reproductive system

**DOI:** 10.1017/erm.2026.10056

**Published:** 2026-06-24

**Authors:** Fangfang Dai, Gongcai Tao, Jie Zhang, Zhimin Deng, Dongyong Yang, Gantao Chen, Yanwei Sha, Tailang Yin

**Affiliations:** 1 Department of Obstetrics and Gynecology, Renmin Hospital of Wuhan University, Wuhan, China; 2 Renmin Hospital of Wuhan University, Wuhan, China; 3 Department of Andrology, Women and Children’s Hospital, School of Medicine, Xiamen University, Xiamen, China

**Keywords:** epitranscriptomic regulation, female reproductive system, m6A modification, pregnancy-related diseases, female endocrine disease

## Abstract

N6-methyladenosine (m6A) is the most abundant internal RNA modification and has emerged as an important regulator of reproductive biology. Dysregulation of m6A regulators has been implicated in pregnancy-related disorders, including recurrent implantation failure, miscarriage, preeclampsia, and gestational diabetes, as well as female reproductive endocrine diseases such as polycystic ovary syndrome, premature ovarian insufficiency, and endometriosis. In this review, we summarize current evidence on the roles of m6A writers, erasers, and readers in these disorders, with particular attention to tissue specificity, model systems, downstream targets. We further discuss major sources of inconsistency across studies, including differences in disease stage, cell type, assay platform, and confounding factors. By integrating mechanistic findings with current limitations in the field, this review provides an updated framework for understanding the biological significance of m6A in female reproductive health and disease.

## Graphical abstract

**Figure 1:**
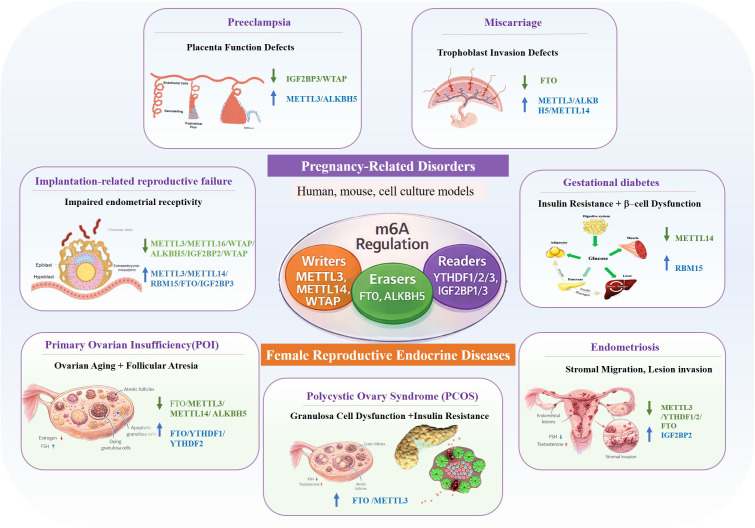
Abstract figure. Overview of m6A RNA modification in pregnancy-related and disorders of the female reproductive system. Central m6A regulators (writers: METTL3/14, WTAP; erasers: FTO, ALKBH5; readers: YTHDF1/2/3, IGF2BP1/3) modulate key reproductive processes. The surrounding panels illustrate specific disorders: preeclampsia, miscarriage, implantation failure, gestational diabetes, POI, PCOS and endometriosis, highlighting how altered m6A regulation affects placental function, trophoblast invasion, endometrial receptivity, ovarian function and granulosa cell or stromal behaviour. **Abbreviation:** gestational diabetes mellitus (GDM); polycystic ovary syndrome (PCOS); N (6)-methyladenosine (m6A); brain-derived neurotrophic factor (BDNF); progesterone receptor (PGR); granulosa cells (GCs); primary ovarian insufficiency (POI); recurrent implantation failure (RIF)

## Introduction

Female reproductive health depends on precisely coordinated molecular, endocrine, immune and metabolic regulation across the ovaries, endometrium, placenta, and early embryo (Ref. 1). Disruption of these processes contributes to a broad range of pregnancy-related disorders and endocrine diseases of the reproductive system, including recurrent implantation failure (RIF), preeclampsia (PE), miscarriage, gestational diabetes mellitus (GDM), polycystic ovary syndrome (PCOS), primary ovarian insufficiency (POI), endometriosis and adenomyosis (Refs. 2, 3). Although these conditions differ in clinical presentation and pathophysiological features, many involve abnormal gene regulation, altered cellular differentiation, inflammatory imbalance, metabolic dysfunction and defective tissue remodelling.

Among the epigenetic and post-transcriptional mechanisms that regulate these processes, N6-methyladenosine (m6A) modification has attracted increasing attention (Refs. 4, 5). m6A is the most prevalent internal modification of eukaryotic messenger RNA and is dynamically regulated by methyltransferases (‘writers’), demethylases (‘erasers’) and m6A-binding proteins (‘readers’) (Refs. 6, 7). Through coordinated regulation of RNA splicing, export, translation, decay and stability, m6A influences cell fate decisions, developmental transitions, stress responses and tissue homeostasis (Refs. 6, 8). An increasing number of studies suggest that abnormal m6A regulation contributes to diverse reproductive phenotypes, including impaired endometrial receptivity, trophoblast dysfunction, ovarian ageing, granulosa-cell injury, lesion invasion and endocrine-metabolic disturbance (Refs. 9, 10).

However, interpretation of the current literature remains challenging. Findings are often derived from a mixture of human clinical samples, animal models and cell-based experiments, and these different lines of evidence are not always clearly separated (Ref. 11). In addition, reported changes in the m6A regulators may vary depending on tissue source, cell type, disease stage, hormonal environment, hypoxic exposure, metabolic state and analytical platform (Refs. 11, 12). As a result, the same regulator may appear protective in one biological context but pathogenic in another. This context dependence is a recurring theme across the field and is essential for interpreting apparently contradictory results.

In this review, we summarize current evidence on the role of m6A modification in pregnancy-related disorders and female reproductive endocrine diseases, with particular emphasis on molecular mechanisms, biological context and major interpretive challenges. Rather than treating all reported associations as equivalent, we distinguish, where possible, between human, animal, and cell-based evidence; between causal and associative findings; and between broadly reproducible observations and more preliminary mechanistic reports. We also discuss major limitations and future priorities for this rapidly evolving field.

## Overview of m6A modification and its regulatory machinery

m6A modification is a reversible and dynamic RNA modification that occurs primarily in the consensus RRACH motif and is enriched around stop codons, within long internal exons, and in 3′ untranslated regions (Ref. 13). This modification is installed by a methyltransferase complex composed mainly of METTL3, METTL14, WTAP, RBM15/15B, VIRMA (KIAA1429), ZC3H13 and related cofactors. m6A marks can be removed by the demethylases FTO and ALKBH5, whereas reader proteins such as YTHDF1/2/3, YTHDC1/2, IGF2BP1/2/3 and ELAVL1 interpret these marks and mediate downstream effects on RNA fate (Ref. 6).

Through this writer–eraser–reader system, m6A regulates multiple aspects of RNA metabolism, including pre-mRNA splicing, nuclear export, transcript stability, translation efficiency and RNA decay (Refs. 14, 15, 16). These functions allow m6A to participate in dynamic biological transitions, including cell proliferation and differentiation, stress adaptation, developmental reprogramming and tissue remodeling (Ref. 17). In the reproductive system, such processes are especially relevant because ovarian folliculogenesis, endometrial receptivity, placental development, immune tolerance and embryo–maternal communication all require tightly controlled gene expression programs (Ref. 18).

Importantly, many m6A regulators also exert m6A-independent functions (Refs. 19, 20). Therefore, the biological effects attributed to a given writer, eraser or reader cannot always be assumed to result exclusively from canonical m6A signalling. This issue is especially relevant in reproductive disease models, where many studies infer mechanisms primarily from expression changes or gain-/loss-of-function phenotypes without fully dissecting whether the observed effects are mediated directly through m6A-dependent RNA regulation (Ref. 21). Accordingly, interpretation of the literature requires caution and careful attention to experimental design.

## Role of m6A modification in pregnancy-related disorders

### m6A and implantation-related reproductive failure

Successful implantation requires precise coordination among embryo development, endometrial receptivity, hormonal responsiveness, immune adaptation and uterine remodelling (Ref. 22). Increasing evidence suggests that dysregulated m6A modification contributes to implantation-related reproductive failure (Ref. 23). However, the available studies span distinct biological contexts, including infertility-associated endometrial dysfunction (Ref. 24), RIF (Ref. 25), and recurrent pregnancy loss-related endometrial abnormalities (Ref. 9). Importantly, current evidence is deriveds from a mixture of human endometrial samples, mouse uterine models and mechanistic tissue- or cell-based experiments, and these different lines of evidence should be interpreted separately.

Among the core m6A writers, METTL3 illustrates the context dependence of this field particularly well. In a uterus-specific loss-of-function mouse model, METTL3 deficiency disrupts endometrial receptivity and causes infertility by disturbing the balance between oestrogen and progesterone signalling, supporting a causal and largely protective role for METTL3 in uterine function (Ref. 26). In contrast, a human endometrial study reported elevated METTL3 expression and increased global m6A levels in RIF, with excessive METTL3 promoting m6A-dependent decay of *HOXA10* mRNA, thereby reducing the expression of a key implantation-supportive gene and impairing embryo implantation (Ref. 25). Similarly, uterine METTL14 deficiency has been shown to compromise implantation, largely through aberrant ER-*α* activation and dysregulated innate immune signaling (Ref. 27).

Taken together, these findings indicate that m6A dysregulation is mechanistically relevant for understanding implantation failure, but its biological significance depends strongly on context. Human observational studies primarily identify associations, whereas conditional mouse models provide stronger evidence for causality. Apparent discrepancies across studies may reflect differences in tissue composition, timing of sampling within the implantation window, hormonal exposure, patient heterogeneity and the analytical platforms used to assess m6A. Therefore, rather than supporting a simple uniformly pathogenic or protective role, current evidence favours a model in which the effects of METTL3 and METTL14 are highly stage, tissue, and dosage dependent. From a clinical perspective, m6A-related alterations may eventually help refine the assessment of endometrial receptivity, but current evidence remains insufficient to support diagnostic or therapeutic application.

### m6A and preeclampsia

PE is a pregnancy-specific hypertensive disorder in which defective trophoblast invasion and placental maladaptation are central pathological features (Ref. 28). Current evidence for m6A dysregulation in PE comes from human placental tissues, hypoxia-treated trophoblast models and a limited number of animal studies (Ref. 29). Across these studies, placental m6A levels are often reported to be elevated (Ref. 30), although the functional significance of individual m6A regulators varies substantially according to the pathway examined and the experimental context.

METTL3 illustrates this context dependence particularly well. In placental tissues from pregnancies with PE and in trophoblast models, increased METTL3 expression has been associated with elevated m6A methylation and trophoblast dysfunction, suggesting a pathogenic role in some settings (Ref. 30). In contrast, other studies have shown that METTL3-mediated m6A can increase circSETD2 expression, suppress miR-181a-5p, elevate MCL1 levels and thereby promote trophoblast proliferation, invasion and survival, implying a protective or compensatory role under different conditions (Ref. 31). Similarly, METTL14 and IGF2BP3 have been reported to enhance circPAPPA2-related signaling and trophoblast invasion, further suggesting that some m6A regulators may act to buffer, rather than directly drive, placental dysfunction in specific biological contexts (Ref. 32). Among the erasers, ALKBH5 is frequently upregulated in placentas with PE and in hypoxia-treated trophoblasts, but its downstream consequences appear to depend on the target transcript and signaling pathway involved. ALKBH5-mediated demethylation has been linked to PPARG- and PLAC8-related pathways, with reported effects on oxidative stress, trophoblast invasion and migration (Refs 33, 34).

Taken together, the available literature supports a mechanistic role for m6A dysregulation in PE, but whether these changes represent primary pathogenic drivers or secondary responses to hypoxia, oxidative stress and placental injury remains unresolved. Important confounding factors include gestational age at sampling, early- versus late-onset disease, clinical severity, maternal metabolic status and differences between bulk placental tissue and isolated trophoblast systems. Accordingly, m6A regulators should currently be regarded as mechanistically relevant candidates rather than clinically validated biomarkers or therapeutic targets in PE.

### m6A and abortion

Spontaneous abortion is the most common complication during the first trimester, with an incidence of 10%–15% (Ref. 35). Increasing evidence suggests that aberrant m6A modification contributes to impaired trophoblast function and pregnancy loss (Ref. 36).

Elevated ALKBH5 expression has been detected in villous tissues from patients who underwent an abortion, where it destabilizes CYR61 mRNA, suppresses trophoblast invasiveness and promotes pregnancy loss (Ref. 37). Conversely, recurrent spontaneous abortion has been associated with reduced ALKBH5 expression in extravillous trophoblasts. Under hypoxic conditions, ALKBH5 undergoes nuclear-to-cytoplasmic redistribution, resulting in insufficient demethylation of SMAD1/SMAD5 transcripts. This impairs their translation, downregulates MMP9 and ITGA1 expression and ultimately restricts trophoblast invasion (Ref. 38). Chorionic villi from patients who underwent a spontaneous abortion also show decreased expression of FTO, IGF2BP1 and IGF2BP2, along with increased levels of METTL3 and WTAP. Downregulation of FTO disrupts immune tolerance and angiogenesis at the maternal–foetal interface, inducing oxidative stress and aberrant methylation that contribute to miscarriage (Ref. 39). In contrast, other studies report elevated FTO protein in recurrent abortion, where it inhibits trophoblast proliferation and invasion via repression of the MEG3-TGF-*β* pathway (Ref. 40). These discrepancies highlight the complexity of FTO’s role, which may vary by gestational stage, tissue type and pathological context.

Collectively, spontaneous abortion involves dysregulated m6A homeostasis, including ALKBH5-mediated demethylation, FTO-dependent signalling and altered expression of m6A readers and writers. These changes converge on pathways that regulate trophoblast invasion, immune tolerance and angiogenesis. Further mechanistic studies are required to reconcile these different findings and clarify the potential clinical relevance of m6A regulators in miscarriage prevention.

### m6A and gestational diabetes

GDM is a pregnancy-specific metabolic disorder that threatens maternal and foetal health, and growing evidence implicates m6A modification in its pathogenesis (Ref. 41). In placental tissues from GDM patients, METTL14 expression is downregulated, leading to reduced global m6A levels (Ref. 41). This reduction predominantly affects the 3′-UTR and CDS of BAMBI, resulting in enhanced TGF-*β* signaling and suppressed Wnt signaling, collectively impairs *β*-cell activity and insulin secretion, thereby exacerbating insulin resistance (Ref. 41). Conversely, experimental overexpression of METTL14 suppresses lncRNA XIST, activates the miR-497-5p/FOXO1 pathway and promotes trophoblast proliferation and migration, ultimately ameliorating GDM progression in rat models (Ref. 42). In foetal liver samples from GDM mice, m6A levels are elevated, accompanied by increased RBM15 expression. Functional studies indicate that RBM15 regulates CLDN4 expression via m6A modification, reducing insulin sensitivity and aggravating insulin resistance (Ref. 43).

Together, these findings highlight the different roles of m6A regulators in GDM. Placental METTL14 deficiency disrupts signaling pathways essential for insulin homeostasis, whereas its overexpression exerts protective effects. In contrast, foetal liver m6A elevation mediated by RBM15 contributes to insulin resistance. These results underscore the pivotal role of m6A in GDM pathogenesis and suggest that precise regulation of m6A modulators may advance our understanding of disease development and progression.

### m6A and other pregnancy-related diseases

In human endometrial tissues from women with infertility or recurrent pregnancy loss, dysregulated expression of several m6A regulators has been reported, including decreased METTL16 and WTAP, and increased ALKBH5 and IGF2BP2 (Ref. 9). During normal pregnancy, uterine m6A levels dynamically increase across gestation, suggesting that m6A contributes to uterine remodelling, implantation and maintenance of pregnancy (Ref. 9). In addition, METTL3 has been shown to be essential for murine oocyte maturation and maternal-to-zygotic transition, highlighting a critical role for m6A-dependent regulation in female germ-cell function and early embryonic development (Ref. 44). Beyond infertility, maternal psychological stress also impacts m6A regulation. Women experiencing prenatal maternal psychological distress (PMPD) in the third trimester exhibit elevated placental m6A methylation, correlating with reduced gestational age and birth weight (Ref. 45). Similarly, fear-induced stress in pregnant rats upregulates METTL3, METTL14 and WTAP, while downregulating FTO, resulting in globally increased placental m6A levels and impaired placental function (Ref. 46).

Collectively, aberrant m6A regulation contributes to diverse pregnancy-related complications, including infertility-associated uterine dysfunction, stress-induced placental alterations and adverse birth outcomes (Table 1). However, several questions remain unresolved, including whether altered m6A patterns are causal or secondary, how m6A interacts with endocrine and metabolic signals throughout gestation and how confounders such as maternal age, obesity and treatment exposure influence outcomes. Moreover, many m6A regulators also have m6A-independent functions, which are poorly characterized in reproductive disorders. Addressing these gaps is essential to clarify the biological significance and translational potential of m6A in pregnancy-related diseases and may uncover novel biomarkers or therapeutic targets.

**Table 1.** Functions of m6A modification in pregnancy-related disorders Table 1. long description.

Disease	Tissue/Cell	Detection method	m6A level	m6A-related molecule expression	Main findings/functions	References
Infertility/pregnancy-related uterine remodelling	Mouse pregnancy uterus; human endometrial tissues from infertility or recurrent pregnancy loss datasets	LC–MS/MS; qPCR; Western blot; IHC; bioinformatic analysis	Up	METTL16(−), WTAP(−), ALKBH5(+), IGF2BP2(+) in human infertility-related endometrial datasets	Uterine m6A levels increase dynamically across gestation in mice. Dysregulated expression of several m6A regulators in infertility-related human endometrium suggests a role for m6A in uterine remodelling, implantation and pregnancy maintenance.	(Ref. 9)
Recurrent implantation failure	PGR-Cre mouse; METTL3 conditional knockout mouse	m6A-seq; m6A-RIP-qPCR	NA	METTL3(−)	METTL3 deficiency increases the stability of oestrogen-responsive transcripts (e.g., *Elf3* and *Celsr2*) and disrupts progesterone-responsive gene expression, thereby causing oestrogen dominance, progesterone resistance and impaired uterine receptivity.	(Ref. 26)
Recurrent implantation Failure	Human endometrial tissue	Colorimetric m6A quantification; dot blot; MeRIP	Up	METTL3(+); HOXA10(−)	METTL3-mediated m6A methylation accelerates HOXA10 mRNA decay, reduces HOXA10 expression, and impairs embryo implantation.	(Ref. 25)
Preeclampsia	Human placental villous tissue, human primary trophoblast cells	MeRIP-Seq	Up	METTL3 (+)	Increased METTL3 expression and elevated m6A methylation are associated with trophoblast dysfunction in preeclampsia.	(Ref. 30)
Preeclampsia	Human placenta, human primary trophoblast cells	Colorimetric m6A quantification strategy MeRIP-PCR	Up	IGF2BP3 (−)	IGF2BP3 recognizes m6A-modified circPAPPA2 and enhances its stability, thereby promoting trophoblast invasion.	(Ref. 32)
Preeclampsia	Preeclampsia rat model; HTR8/SVneo cells	Colorimetric m6A quantification; MeRIP-PCR	Down	ALKBH5 (+)	Inhibition of ALKBH5 increases m6A modification of PPARG mRNA and alleviates oxidative stress and apoptosis through the PPARG/KDM3B/ALCAM/Wnt/*β*-catenin axis.	(Ref. 33)
Preeclampsia	Preeclampsia rat model, HTR8/SVneo	Colorimetric m6A quantification strategy MeRIP-PCR	NA	METTL3 (+)	METTL3 overexpression increases circSETD2 expression, suppresses miR-181a-5p, elevates MCL1 and promotes trophoblast proliferation and invasion while reducing apoptosis.	(Ref. 31)
Preeclampsia	HTR8/SVneo	MeRIP-PCR	NA	ALKBH5 (+)	ALKBH5 reduces m6A levels on PLAC8 mRNA, thereby increasing PLAC8 expression and enhancing hypoxia-induced trophoblast invasion and migration.	(Ref. 34)
Preeclampsia	Human placenta; HTR8/SVneo cells	Colorimetric m6A quantification	Down	WTAP (−)	WTAP regulates HMGN3 expression in an m6A-dependent manner and contributes to trophoblast dysfunction in preeclampsia.	(Ref. 47)
Abortion/ miscarriage	human primary trophoblast cells, HTR8/SVneo Villous explant	Colorimetric m6A quantification strategy MeRIP-qPCR	Down	ALKBH5 (+)	ALKBH5 destabilizes CYR61 mRNA, suppresses trophoblast invasion and contributes to miscarriage.	(Ref. 37)
Abortion/ miscarriage	Human primary trophoblast cells; HTR8/SVneo cells; villous explants	Dot blot; RIP; MeRIP	Up	FTO (−) METTL3 (+)	Reduced FTO expression is associated with abnormal methylation, oxidative stress, impaired immune tolerance, and defective angiogenesis at the maternal–foetal interface.	(Ref. 39)
Abortion/ miscarriage	Human chorionic villous tissue	qPCR m6A-IP	Up	ALKBH5(+) ALKBH5 altered	Under hypoxic conditions, altered ALKBH5-mediated demethylation of SMAD1/5 affects trophoblast invasion-related pathways.	(Ref. 38)
Abortion/ miscarriage	Human trophoblast tissue, Swan 71 cell	MeRIP	Up	METTL14 (+)	BPDE exposure induces MXD1-dependent METTL14 transcription and increases m6A modification of lnc-HZ01, thereby inhibiting trophoblast proliferation and promoting miscarriage.	(Ref. 48)
Abortion/ miscarriage	Human trophoblast tissue, human primary trophoblast cells	Dot blot; MeRIP-PCR	Down	FTO (+)	FTO reduces m6A modification of MEG3 and disrupts the MEG3/TGF-*β* axis, thereby contributing to recurrent spontaneous abortion.	(Ref. 40)
Abortion/ implantation failure-related injury	HTR8/SVneo cells; mouse model	MeRIP-PCR; LC–MS/MS	Down	METTL3 (+)	Melatonin protects against LPS-induced implantation failure and abnormal pregnancy through the MTNR1B/m6A pathway.	(Ref. 49)
Gestational diabetes mellitus	Human placenta	MeRIP-seq; MazF-qPC	Down	METTL14 (−)	Downregulation of METTL14 reduces m6A modification in the 3′-UTR and CDS of *BAMBI*, thereby enhancing TGF-*β* signalling and suppressing Wnt signalling, with downstream impairment of *β*-cell function and insulin homeostasis.	(Ref. 41)
Gestational diabetes mellitus	Mouse serum and liver tissue; LO2 cells; primary mouse liver cells	qPCR; MeRIP; dot blot	Up	RBM15 (+)	RBM15 promotes CLDN4 expression through m6A modification, reduces insulin sensitivity and exacerbates insulin resistance.	(Ref. 43)
Gestational diabetes mellitus	Human placenta; HTR8/SVneo cells	MeRIP-PCR	Down	METTL14 (−)	METTL14-mediated silencing of XIST activates the miR-497-5p/FOXO1 axis, promotes trophoblast proliferation and migration and mitigates GDM-related phenotypes.	(Ref. 42)
Gestational diabetes mellitus	Human primary trophoblast cells; umbilical cord blood-related context	EpiQuik m6A RNA Methylation Quantification Kit	Up	NA	Increased m6A levels are observed in GDM-related samples, and hesperidin reduces m6A levels and improves trophoblast cell activity under LPS/high-glucose conditions.	(Ref. 50)
Other pregnancy-related diseases	Human placenta	MeRIP-PCR	Up	NA	Increased prenatal maternal psychological distress is associated with elevated placental m6A methylation and adverse birth outcomes.	(Ref. 45)
Other pregnancy-related diseases	Placenta from fear-stress rat model	MeRIP-PCR	Up	METTL3(+), METTL14 (+), WTAP (+); FTO (−)	Fear stress during pregnancy alters placental m6A-modifying enzymes and increases placental m6A methylation, indicating stress-related placental epitranscriptomic dysregulation.	(Ref. 46)

*Note:* RIF: recurrent implantation failure; LC–MS/MS: liquid chromatography–tandem mass spectrometry; qPCR: quantitative polymerase chain reaction; IHC: immunohistochemistry; MeRIP: methylated RNA immunoprecipitation; METTL3: methyltransferase-like 3; METTL14: methyltransferase-like 14; WTAP: Wilms tumour 1-associated protein; ALKBH5: AlkB homologue 5, RNA demethylase; FTO: fat mass and obesity-associated protein; IGF2BP2/3: insulin-like growth factor 2 mRNA-binding protein 2/3; HOXA10: homeobox A10; PPARG: peroxisome proliferator-activated receptor gamma; KDM3B: lysine demethylase 3B; ALCAM: activated leukocyte cell adhesion molecule; PLAC8: placenta-associated 8; HMGN3: high mobility group nucleosome-binding domain-containing protein 3; CYR61: cysteine-rich angiogenic inducer 61; SMAD1/5: SMAD family member 1/5; lnc-HZ01: long non-coding RNA HZ01; MEG3: maternally expressed 3; TGF-*β*: transforming growth factor beta; RBM15: RNA binding motif protein 15; CLDN4: claudin 4; XIST: X inactive specific transcript; FOXO1: forkhead box O1.

**Table 2.** Functions of m6A modification in female reproductive endocrine diseases Table 2. long description.

Disease	Tissue/Cell	Detection method	m6A level	m6A-related molecule expression	Main findings/functions	References
PCOS	KGN cells (human granulosa-like tumour cell line)	MeRIP-qPCR	Down	FTO (+)	FTO reduces m6A modification of FLOT2 mRNA, increases FLOT2 mRNA stability and promotes granulosa-cell dysfunction, including altered proliferation/apoptosis and insulin resistance.	(Ref. 52)
PCOS	Mouse ovary; granulosa cells	EpiQuik m6A RNA methylation quantification kit; MeRIP-qPCR	Up	METTL3 (+)	Reduced butyrate associated with gut microbiota dysbiosis is linked to increased ovarian METTL3 expression, enhanced m6A modification of FOSL2 and ovarian inflammatory changes in PCOS-related models.	(Ref. 53)
PCOS	Human primary luteinized granulosa cells	Colorimetric m6A quantification; MeRIP-qPCR; MeRIP-seq	Up	NA	Altered m6A modification is associated with increased FOXO3 expression in luteinized granulosa cells from women with PCOS after controlled ovarian hyperstimulation.	(Ref. 54)
POI/ovarian aging	Human follicular fluid and granulosa cells; mouse ovaries	ELISA; qRT-PCR; Western blot; IHC; colorimetric m6A quantification	Up	FTO (−)	Across human follicular fluid, granulosa cells and mouse ovaries, reduced FTO expression is associated with increased global m6A levels during ovarian ageing.	(Ref. 55)
POI-related ovarian injury	Human granulosa cells; mouse ovaries	Colorimetric m6A quantification; qPCR; Western blot	Up	multiple methyltransferases (+); FTO (−); m6A effectors/readers (−); ALKBH5 (no obvious change)	Cyclophosphamide increases global m6A levels in granulosa cells and mouse ovaries in a time- and concentration-dependent manner, with increased methyltransferase expression and reduced FTO expression, suggesting enhanced RNA methylation during ovarian injury.	(Ref. 56)
POI	COV434 human granulosa tumour cell line	Dot blot; LC-MS/MS; MeRIP-qPCR	Down	ALKBH5 (−)	In a VCD-associated POI model, reduced ALKBH5 is linked to altered YAP-related m6A signalling and ovarian injury.	(Ref. 57)
POI	Granulosa cells; rat POI model	RT-qPCR; Western blot; ROS staining; SA-beta-gal; JC-1; TEM; OCR; ATP assay	NA	FTO-circBRCA1/miR-642a-5p/FOXO1 axis altered	FTO stabilizes circBRCA1 through m6A demethylation, thereby improving mitochondrial function and reducing oxidative stress-related granulosa-cell damage in POI models.	(Ref. 58)
POI	Follicular theca cells; granulosa cells; mouse model	Genetic knockout; RT-qPCR; Western blot; MeRIP; functional assays	NA	METTL3 (−)	METTL3 deficiency in theca cells reduces pri-miR-21 m6A modification, decreases mature miR-21-5p, increases IL-1beta secretion, promotes granulosa-cell apoptosis and reduces oestradiol synthesis, leading to a POI-like phenotype.	(Ref. 59)
Endometriosis	Ectopic lesions; human endometrial stromal cells (HESCs)	Colorimetric m6A quantification; dot blot; MeRIP-qPCR	Down	METTL3 (−)	Reduced METTL3 lowers pri-miR-126 m6A modification, impairs pri-miR-126 maturation and promotes stromal-cell migration and invasion.	(Ref. 60)
Endometriosis	Ectopic endometrium; primary endometrial stromal cells	Colorimetric m6A quantification; dot blot; MeRIP-qPCR	Down	METTL3 (−); YTHDF2 (−)	METTL3-mediated m6A facilitates YTHDF2-dependent regulation of SIRT1 mRNA; reduced METTL3/m6A signalling suppresses stromal-cell senescence and favours lesion progression.	(Ref. 61)
Adenomyosis	Human endometrium and myometrium	Colorimetric m6A quantification; MS/MS	Down	METTL3 (−); YTHDF1/2 (−)	Abnormal expression and activity of m6A regulators in endometrium and myometrium may contribute to the development of adenomyosis.	(Ref. 62)
Endometriosis	Ectopic endometrium; primary endometrial stromal cells	Colorimetric m6A quantification; MeRIP-PCR	Up	FTO (−)	FTO overexpression enhances ATG5 expression in an m6A-dependent manner, suppresses PKM2-driven glycolysis and inhibits stromal-cell proliferation and invasion, supporting a protective role in ectopic stromal cells.	(Ref. 63)
Endometriosis (ovarian)	Primary endometrial stromal cells	Dot blot; MeRIP-PCR	Up	IGF2BP2 (+)	The m6A reader IGF2BP2 stabilizes MEIS2 and GATA6 mRNAs and promotes proliferation, migration, and invasion of ectopic stromal cells in ovarian endometriosis.	(Ref. 64)
Endometriosis (ovarian)	Ovarian endometriosis tissue; THESCs	m6A transcriptome profiling; qRT-PCR; in vitro functional assays	Down (global)	ALKBH5-related DIO3OS regulation	ALKBH5 regulates lncRNA DIO3OS through m6A modification and promotes stromal-cell invasion and migration in ovarian endometriosis.	(Ref. 65)

*Note:* NA: not available/not reported; Up: increased global m6A level or upregulated expression; Down: decreased global m6A level or downregulated expression; PCOS: polycystic ovary syndrome; KGN: human granulosa-like tumour cell line; FTO: fat mass and obesity-associated protein; FLOT2: flotillin 2; METTL3: methyltransferase-like 3; FOSL2: FOS-like 2; FOXO3: forkhead box O3; POI: primary ovarian insufficiency; ALKBH5: AlkB homolog 5 RNA demethylase; VCD: 4-vinylcyclohexene diepoxide; YAP: Yes-associated protein; circBRCA1: circular BRCA1; SIRT1: sirtuin 1; ATG5: autophagy related 5; PKM2: pyruvate kinase M2; IGF2BP2: insulin-like growth factor 2 mRNA-binding protein 2; MEIS2: Meis homeobox 2; GATA6: GATA binding protein 6.

## Role of m6A modification in female endocrine diseases

Endocrine diseases of the female reproductive system include PCOS, POI and endometriosis. m6A modification is crucial for regulating gene expression, cell differentiation and metabolic processes (Ref. 51). Numerous studies have highlighted the involvement of m6A modification in the development and progression of female endocrine diseases (Table 2).

### m6A and polycystic ovarian syndrome

PCOS is a heterogeneous endocrine-metabolic disorder characterized by hyperandrogenism, ovulatory dysfunction, insulin resistance and granulosa cell abnormalities (Ref. 66). Current m6A-related evidence in PCOS is derived mainly from granulosa cells, follicular fluid and experimental ovarian models, with relatively limited information on systemic metabolic tissues (Ref. 67). Thus, most available studies address local ovarian mechanisms rather than the full multisystem biology of PCOS.

FTO is one of the most consistently implicated m6A regulators in PCOS. Elevated FTO expression has been reported in follicular fluid and granulosa cells from patients with PCOS, where it is associated with reduced global m6A levels and higher androgen levels (Ref. 68). Mechanistically, FTO promotes FLOT2 expression by reducing m6A modification on FLOT2 mRNA and increasing its stability, thereby contributing to granulosa-cell dysfunction, including enhanced proliferation, reduced apoptosis and insulin resistance (Ref. 52). In addition, DHT exposure increases FTO expression and suppresses m6A levels, supporting a mechanistic link between hyperandrogenism and epitranscriptomic dysregulation in PCOS (Ref. 68). METTL3 has also been implicated, but its role appears more context specific. Some studies suggest that METTL3-mediated m6A promotes inflammatory and metabolic dysfunction in granulosa cells, for example, through CD36-related pathways (Ref. 69), whereas others indicate that METTL3 silencing can alter ferroptosis- and fibrosis-related responses in experimental PCOS models (Ref. 70).

These divergent findings may reflect differences in the model induction method, cell state, inflammatory milieu and readouts used to define disease phenotype. Overall, current evidence supports involvement of m6A in ovarian aspects of PCOS, but it remains unclear whether these alterations are causal drivers of disease initiation or secondary adaptations to androgen excess and metabolic stress. Future work should more explicitly integrate ovarian findings with obesity, insulin resistance and whole-body metabolic phenotypes before translational conclusions are drawn.

### m6A and primary ovarian insufficiency

POI is defined by loss of ovarian function before the age of 40 years and is characterized by menstrual disturbance, hypoestrogenism, infertility and long-term health consequences (Ref. 71). Evidence linking m6A dysregulation to POI comes from studies of ovarian ageing, toxicant-induced ovarian injury, granulosa cells and ovarian cell lines (Ref. 72). These contexts are related but not identical, and this distinction is important when interpreting mechanistic findings.

Age-related reduction in FTO is among the more reproducible observations in ovarian ageing- and POI-related settings. Clinical and animal studies have shown that FTO expression decreases in ovarian samples from patients with POI and in POI mouse models, accompanied by increased global m6A levels. Similarly, in human follicular fluid, granulosa cells and aging mouse ovaries, FTO expression declines with age, whereas total m6A levels increase, supporting an association between reduced FTO and age-related ovarian dysfunction (Refs 55, 73). Mechanistically, FTO downregulation in granulosa cells promotes ovarian aging by increasing m6A modification of FOS-related transcripts, reducing their decay and enhancing FOS expression (Ref. 74). In another context, FTO has also been reported to stabilize circBRCA1 through m6A demethylation and improve mitochondrial function via the circBRCA1/miR-642a-5p/FOXO1 axis in a POI-related oxidative stress model (Ref. 58). Together, these findings suggest that FTO is an important but context-dependent regulator in ovarian aging and POI.

Additional evidence from toxicant- and drug-induced ovarian injury models indicates that disrupted m6A homeostasis may contribute to POI-related phenotypes. In cyclophosphamide (CTX)-exposed granulosa cells and mouse ovaries, global m6A levels increase in a time- and concentration-dependent manner, accompanied by upregulation of multiple methyltransferases and downregulation of FTO, whereas ALKBH5 appears largely unchanged (Ref. 56). In contrast, in a 4-vinylcyclohexene diepoxide (VCD)-induced rat model of POI, total m6A levels were decreased, and altered ALKBH5-mediated YAP m6A regulation is implicated in ovarian injury (Ref. 57). More direct mechanistic evidence comes from a theca-cell-specific METTL3 deficiency model, in which reduced m6A modification of pri-miR-21 impairs miR-21-5p maturation, increases IL-1*β* secretion and secondarily promotes granulosa-cell apoptosis and reduced oestradiol synthesis, ultimately leading to a POI-like phenotype (Ref. 59). Together, these findings support a contributory role of m6A dysregulation in POI while also highlighting substantial heterogeneity across toxicant-induced, ageing-related, and cell-type-specific models.

Collectively, aberrant m6A regulation contributes to ovarian ageing and POI through both intrinsic (age-related decline in FTO, dysregulated METTL3) and extrinsic (toxicant-induced suppression of FTO or ALKBH5) mechanisms. These findings identify FTO, METTL3 and ALKBH5 as promising mechanistic candidates in ovarian ageing and POI, although their value as clinical biomarkers or therapeutic targets remains to be established.

### m6A and endometriosis

Endometriosis is a chronic inflammatory disorder characterized by ectopic implantation and survival of endometrial-like tissue, with important contributions from stromal invasion, immune dysregulation, altered metabolism and progesterone resistance (Ref. 75). Compared with several other gynaecologic disorders, the literature on m6A in endometriosis is relatively more extensive (Ref. 61); however, the findings are also particularly heterogeneous because studies differ substantially in lesion subtype, tissue source, cellular context and analytical platform.

In ectopic lesions and stromal-cell models, METTL3 is generally downregulated, and its loss promotes migration and invasion by impairing DGCR8-dependent maturation of pri-miR-126 and by altering the YTHDF2/SIRT1/FOXO3a axis, thereby favouring lesion progression (Refs 60, 61). In contrast, within the immune microenvironment, METTL3 activation has been linked to lactate-associated M2 macrophage polarization through the Trib1/ERK/STAT3 pathway, which may also facilitate lesion growth (Ref. 76). Other m6A regulators show similar context specificity. FTO appears to exert a protective effect on ectopic stromal cells by suppressing glycolysis, proliferation and metastasis through the ATG5/PKM2 axis (Ref. 63). In ovarian endometriosis, ALKBH5 promotes stromal-cell invasion and migration through m6A-dependent regulation of lncRNA DIO3OS, whereas IGF2BP2 enhances proliferation, migration and invasion by stabilizing MEIS2 and GATA6 transcripts (Refs. 64, 65).

Overall, current evidence supports a mechanistic involvement of m6A dysregulation in endometriosis; however, the functional impact of individual regulators appears highly context dependent, varying with lesion subtype, cellular compartment, and the local metabolic-immune microenvironment. Notably, most available data are derived from stromal-cell systems, ovarian endometriotic lesions and selected immune models. As such, the existing evidence remains insufficient to support uniform biomarker development or therapeutic targeting strategies. Future studies should therefore prioritize systematic dissection of epithelial-, stromal-, and immune-cell-specific m6A regulation across different disease stages and anatomical subtypes.

m6A regulators also provide important insights into other endocrine disorders of the female reproductive system, including PCOS and POI, but key gaps remain. In PCOS, studies are largely restricted to granulosa cells, and it remains unclear whether m6A dysregulation contributes to systemic metabolic disturbances such as obesity and insulin resistance. The effects of environmental exposures on ovarian function via m6A pathways are also poorly understood. In both PCOS and POI, mechanistic studies are mainly limited to granulosa cell proliferation and apoptosis, and it remains uncertain whether findings can be generalized across diseases or reconciled when inconsistent.

In summary, future work should integrate systemic metabolic profiling, environmental factors, cross-disease comparisons and infertility models. Importantly, many m6A regulators also exhibit m6A-independent functions, which remain largely unexplored and may represent additional mechanisms and therapeutic targets. Integrating both m6A-dependent and m6A-independent roles will be essential for a comprehensive understanding of endocrine diseases of the reproductive system.

## Future perspectives

As the most prevalent internal RNA modification in eukaryotes, m6A has emerged as an important regulator in reproductive endocrinology. Increasing evidence suggests that the same m6A regulator may exert either protective or pathogenic effects depending on disease stage, cell type, hormonal context, and downstream targets. This context dependence remains a major challenge in the field.

Several factors may contribute to this complexity. First, m6A regulators can influence diverse downstream transcripts and signaling pathways, leading to distinct biological outcomes in different cellular contexts. Second, female reproductive endocrine diseases are strongly influenced by hormonal fluctuations, which may reshape the epitranscriptomic landscape. Third, current detection technologies still face limitations in sensitivity, resolution, cost and reproducibility, thereby restricting cross-study comparisons and clinical translation. Future studies should therefore focus on several priorities: clarifying the downstream regulatory networks of m6A factors; analysing large-scale clinical samples to define disease-specific m6A signatures; applying emerging technologies such as single-cell sequencing, nanopore sequencing, CRISPR/Cas9-based functional screening, and advanced computational approaches; investigating the spatiotemporal dynamics of m6A during oocyte maturation, fertilization, and early embryogenesis; and exploring the crosstalk between m6A and other epigenetic mechanisms.

Importantly, most, if not all, m6A writers, erasers, and readers have been reported to exhibit m6A-independent functions. These regulators may participate in gene expression control, protein–protein interactions, chromatin remodelling or signaling pathways independently of their roles in RNA methylation. Therefore, phenotypic effects observed in current studies cannot always be unequivocally attributed to m6A-dependent mechanisms, representing a potential confounding factor in the interpretation of existing literature. Future research should prioritize distinguishing m6A-dependent from m6A-independent effects through refined experimental strategies, such as catalytic-dead mutants, domain-specific functional analyses and integrative multi-omic approaches. Elucidating these non-canonical functions will not only improve mechanistic clarity but may also uncover novel pathways involved in reproductive disease pathogenesis, thereby expanding potential therapeutic targets beyond the m6A modification axis.

## Conclusion

In summary, m6A RNA modification has emerged as a key epitranscriptomic regulator in pregnancy-related disorders and endocrine diseases of the female reproductive system, influencing trophoblast function, endometrial receptivity, ovarian biology and metabolic homeostasis. Current evidence consistently links dysregulated m6A machinery to disease phenotypes although most findings remain context dependent and are derived from a combination of human, animal, and cell-based studies. Importantly, the biological effects of individual m6A regulators vary across cell types, disease stages, and microenvironmental conditions, and may involve both m6A-dependent and m6A-independent mechanisms. Future studies integrating multi-omic approaches and well-defined clinical cohorts will be essential for clarifying causality and enabling the translation of m6A biological features into clinically relevant biomarkers and therapeutic strategies.

## Long descriptions

### Table 1. Long description

The table consists of 21 rows of data categorized by disease type.

1. Infertility/pregnancy-related uterine remodelling: Studied in mouse uterus and human endometrial tissues using L C-M S/M S and qPCR. m 6 A levels are Up. Expression shows METTL16 minus, WTAP minus, ALKBH5 plus, and IGF2BP2 plus. Findings indicate dynamic m 6 A increases across gestation.

2. Recurrent implantation failure: Studied in METTL3 knockout mice. m 6 A level is N A. METTL3 minus expression increases stability of estrogen-responsive transcripts like Elf3 and Celsr2.

3. Recurrent implantation failure: Studied in human endometrial tissue. m 6 A levels are Up. METTL3 plus and HOXA10 minus expression accelerates HOXA10 m R N A decay.

4. Preeclampsia: Multiple entries show varied results. One entry in human placental tissue shows m 6 A levels Up with METTL3 plus, associated with trophoblast dysfunction. Another entry shows m 6 A levels Down with ALKBH5 plus, where inhibition of ALKBH5 alleviates oxidative stress via the PPARG/KDM3B/ALCAM/Wnt/beta-catenin axis. A third entry shows WTAP minus with m 6 A levels Down, contributing to trophoblast dysfunction.

5. Abortion/miscarriage: Multiple entries. One shows m 6 A levels Down with ALKBH5 plus, destabilizing CYR61 m R N A. Another shows m 6 A levels Up with FTO minus and METTL3 plus, associated with impaired immune tolerance. A third entry shows m 6 A levels Up with METTL14 plus due to B P D E exposure.

6. Gestational diabetes mellitus: Entries show m 6 A levels Down with METTL14 minus, enhancing T G F-beta signaling and suppressing Wnt signaling. Another entry shows m 6 A levels Up with RBM15 plus, reducing insulin sensitivity.

7. Other pregnancy-related diseases: Includes prenatal maternal psychological distress and fear-stress rat models, both showing m 6 A levels Up in the placenta, indicating stress-related epitranscriptomic dysregulation.

Navigate back to Table 1..

### Table 2. Long description

The table is organized into seven columns: Disease, Tissue/Cell, Detection method, m 6 A level, m 6 A-related molecule expression, Main findings/functions, and References.

Key entries include:

* P C O S: In K G N cells, m 6 A levels are down due to F T O (+) expression, which reduces m 6 A modification of F L O T 2 m R N A and promotes granulosa-cell dysfunction. In mouse ovaries, m 6 A levels are up due to M E T T L 3 (+) expression linked to gut microbiota dysbiosis.

* P O I and Ovarian Aging: In human and mouse tissues, m 6 A levels are up while F T O expression is down. Cyclophosphamide-induced injury also increases m 6 A levels via increased methyltransferases and reduced F T O. Conversely, in a V C D-associated model, m 6 A levels are down with reduced A L K B H 5.

* Endometriosis: Multiple studies show m 6 A levels are generally down in ectopic lesions and stromal cells, often associated with reduced M E T T L 3 or Y T H D F 2, promoting cell migration and invasion. However, some specific models show m 6 A levels up when F T O is reduced or I G F 2 B P 2 is increased, affecting S I R T 1 or M E I S 2 pathways.

* Adenomyosis: Shows down-regulated m 6 A levels in human endometrium and myometrium associated with reduced M E T T L 3 and Y T H D F 1 or 2.

Navigate back to Table 2..
